# Group demography affects ant colony performance and individual speed of queen and worker aging

**DOI:** 10.1186/s12862-017-1026-8

**Published:** 2017-08-01

**Authors:** Julia Giehr, Jürgen Heinze, Alexandra Schrempf

**Affiliations:** Zoology/ Evolutionary Biology, University of Regensburg, D-93053 Regensburg, Germany

**Keywords:** Group demography, Social insects, Aging, Task performance, Colony productivity

## Abstract

**Background:**

The performance and fitness of social societies mainly depends on the efficiency of interactions between reproductive individuals and helpers. Helpers need to react to the group’s requirements and to adjust their tasks accordingly, while the reproductive individual has to adjust its reproductive rate. Social insects provide a good system to study the interrelations between individual and group characteristics. In general, sterile workers focus on brood care and foraging while the queen lays eggs. Reproductive division of labor is determined by caste and not interchangeable as, e.g., in social mammals or birds. Hence, changing social and environmental conditions require a flexible response by each caste. In the ant *Cardiocondyla obscurior*, worker task allocation is based on age polyethism, with young workers focusing on brood care and old workers on foraging. Here, we examine how group age demography affects colony performance and fitness in colonies consisting of only old or young workers and a single old or young queen. We hypothesized that both groups will be fully functional, but that the forced task shift affects the individuals’ performance. Moreover, we expected reduced worker longevity in groups with only young workers due to precocious foraging but no effect on queen longevity depending on group composition.

**Results:**

Neither the performance of queens nor that of workers declined strongly with time per se, but offspring number and weight were influenced by queen age and the interaction between queen and worker age. Individual residual life expectancy strongly depended on colony demography instead of physiological age. While worker age affected queen longevity only slightly, exposing old workers to the conditions of colony founding increased their life spans by up to 50% relative to workers that had emerged shortly before colony set-up.

**Conclusions:**

The social environment strongly affected the tempo of aging and senescence in *C. obscurior*, highlighting the plasticity of life expectancy in social insects. Furthermore, colonies obtained the highest reproductive output when consisting of same-aged queens and workers independent of their physiological age. However, workers appeared to be able to adjust their behavior to the colony’s needs and not to suffer from age-dependent restrictions.

**Electronic supplementary material:**

The online version of this article (doi:10.1186/s12862-017-1026-8) contains supplementary material, which is available to authorized users.

## Background

The performance of animal societies relies critically on the traits of individual group members, and the quality of their interactions (e.g. [1–3]). At the same time, social environment and task allocation may strongly affect traits of the individual, including learning capability, susceptibility to diseases, and “personality” [4–7].

Social insects represent a suitable system to study how differences between individual group members and the composition of the group affect the fitness of the group [8, 9]. Insect societies are characterized by reproductive division of labor between queens (in termites also kings) and sterile helpers (workers). In addition, workers of many species specialize on different non-reproductive tasks. While task allocation among workers may be influenced by their experience, morphology, genetic background, and social interactions [10–13], in many species the chronological age of workers plays a major role: younger individuals preferentially engage in brood care in the nest, while older individuals focus on foraging outside and nest defense [14–18].

Nevertheless, the behavioral trajectory is far from unidirectional and can be reversed in response to environmental or social challenges [17, 19–21]. For example, the removal of older nestmates induces young workers to forage precociously and foragers may return to brood care activities if needed. Precocious foraging and reversal to nursing may be associated with an expansion of the behavioral repertoire of individuals [18]. However, studies on honeybees and a few ant species indicate that task reversal may result in weaker individual performance [22–25] and can also affect the individual longevity of workers [26, 27].

Similar to workers, the behavior and productivity of queens may change with age. For example, while young queens of *Temnothorax rugatulus* are capable of solitary founding and independently rearing their first offspring, older queens lose this capability [28]. In *Cardiocondyla obscurior*, the egg laying rate of queens increases with age until shortly before their death and old queens of several ant species [29–31] produce more female sexual offspring than young queens even when provided with the same number of workers. Any disruption of the natural age structure of insect colonies might therefore influence their performance [32], but the effects of behavioral flexibility on the robustness of division of labor and colony fitness remain largely unstudied [20].

Here we take an extreme approach to examine the consequences of age demography on colony productivity and individual life expectancies of queens and workers in the ant *Cardiocondyla obscurior.* In this ant, division of labor among workers in natural colonies follows a typical age polyethism ([33]; AS, unpublished results). In a full-factorial design we set up experimental colonies with either freshly eclosed or 12 week old queens, and either freshly eclosed or 12 week old workers. This forced workers to forage prematurely or to reverse to nursing to sustain the colony and exposed queens of a given age to a novel social environment. We investigated whether manipulated workers were less efficient due to presumed behavioral or physiological constrains (see [20–25]) or whether worker task performance is rather independent of age [34]. Our results show that both colonies with only young or only old workers were able to raise offspring. Yet, colony performance was strongly affected by its age composition. Furthermore, the residual lifespan of queens and workers depended more on group composition than on chronological age. Hence, we can show that colony age demography affects worker task allocation and physiology in ants, as previously shown in honey bees [26, 35–37].

## Methods

### (a) study species and experimental set-up

*C. obscurior* is a small (body length approximately 1.5 mm) myrmicine ant, which human activities have accidentally introduced from its presumed origin in Southeast Asia throughout the tropics and subtropics [38]. Its colonies consist of one or several queens and a few dozen workers [39] and nest in preformed cavities in trees, such as galls, rolled lemon leaves, or aborted coconuts [40, 41]*.* Female sexuals and wingless males are produced year-round and readily mate in the natal nest, resulting in high inbreeding levels [38, 32]. Queens lay one or two eggs per day and live on average for 26 weeks [42]. Workers are completely sterile and have a life expectancy of 12 to 16 weeks [43].

Our study colonies were derived from six split colonies originating from one large laboratory stock colony originally collected in a population introduced to Ilhéus, Brazil. Colonies in Brazil are genetically depauperate and highly inbred [44]. Hence, possible effects due to different genetic “lineages” are reduced and observed differences between crosses are most likely due to physiological processes. Colonies were housed in 9.6 × 9.6 × 3 cm^3^ plastic boxes with a plaster floor and three chambers for the nest cavity, food, and water. They were kept in incubators at near-natural day/night cycles of 12 h 25 °C/ 12 h 24 °C and fed three times per week with cockroaches or fruit flies and honey. The number of individuals, brood items, and dead ants was counted twice per week during the complete experiment.

The experiment consisted of two phases. The purpose of the first phase was to create colonies with “old” workers and queens. In the second phase, these old individuals were used to create experimental colonies with old workers and young queens from the stock colonies and vice versa.

Phase I: 32 experimental colonies were set up within one week with 30 worker pupae, one queen pupa, and one male pupa from split colonies derived from one original stock colony from Bahia, Brazil. To be able to replace dead workers with individuals of similar age at the beginning of phase II, for each stock colony we set up “back-up colonies” with at least 30 worker pupae and several queen pupae depending on availability. Dead pupae or adults were replaced during the first week to maintain the standardized group composition of 30 workers, one queen and one male. All pupae hatched within the first week (week 0); hence the age of nestmates did not differ for more than seven days. Subsequently, colonies were scanned twice a week, egg number was counted, and all newly produced pupae were removed (after caste and number had been recorded) to avoid that young individuals eclosed during this period. Three experimental colonies failed to establish a reproducing colony (two queens died within one week after the first egg was laid, one colony strongly decreased in size until all workers had been dead). These colonies were excluded from further experiments. After twelve weeks, 39% of the workers from the remaining 29 colonies had died and queens were in the middle of their average reproductive lifespan [42]. Twelve-week old workers and queens are referred to as “old.”

**Fig. 1:**
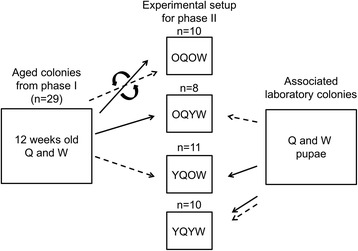
Experimental setup of *Cardiocondyla obscurior* colonies. Dotted lines represent workers, continuous lines queens

Phase II: Twelve weeks after the original colonies had been set up we removed all brood items from the successfully established colonies (*n* = 29, mean ± sd: 7.18 ± 3.2 eggs and 17.5 ± 4.4 larvae, Fig. 1) and added workers from the “back-up colonies” to standardize the initial number of workers per colony to 20. The “old” workers and queens, and pupae from the stock colonies were randomly assigned to create colonies with an old queen and 20 young workers (OQYW, *n* = 8), young queens and 20 old workers (YQOW, *n* = 11), and, by reciprocal exchanges, with old queens and old workers (OQOW, *n* = 10). In addition, new colonies were created with young queens and young workers for comparisons (YQYQ, *n* = 10). We recorded the age of death of queens and counted the number of eggs, larvae, and pupae in the colonies twice per week without removing brood items until all individuals in a colony had died. Ants initially react sensitively to experimental manipulations, which can result in the accidental death of some individuals shortly after colony set-up (e.g., by drowning in honey before the colony was successfully established). Furthermore, several queens disappeared or did not lay any eggs. Hence only 19 of these 29 colonies could be considered for the analysis of lifespans and brood production (OQOW *n* = 5; OQYW *n* = 6, YQOW *n* = 3; YQYW *n* = 5).

### (b) offspring weight

To examine offspring biomass, which might indicate its quality, we pooled six freshly eclosed workers (identified by their lighter color) from three colonies per group, killed them by freezing, and weighed them to the nearest 0.1 microgram (μm) using a fine scale (Sartorius Micro SC2). To check for changes with the age of the colony, we examined the wet weight of the first six produced workers in phase II (after about eight to ten weeks) and of six workers that have been produced ten weeks later. Measurements were taken twice and the mean was used for analysis.

### (c) behavioral observations

To examine behavioral differences between the different treatments we scanned the behavioral status of workers within 34 experimental colonies (OQOW: *n* = 9, OQYW *n* = 7, YQOW *n* = 9, YQYW: *n* = 9) for five or six times per day during the first twelve days of phase II. Workers feeding or being located next to a food source were recorded as foragers as *C. obscurior* workers are too small to measure the quantity of transferred food.

In addition we videotaped the age-dependent motion behavior throughout a worker lifespan. Therefore, we set up three additional colonies with 20 individuals of equal age (see phase I) to avoid any effect of this treatment on the experimental colonies. Two randomly chosen workers of these colonies were separately filmed for 10 min once per week over a 30-weeks period. Due to mortality, sample size decreased with time (see Additional file 1: Figure S1). The recorded videos were analyzed by EthoVision XT 10 software (Noldus, Wageningen).

### (d) data analysis

Statistical analyses were conducted using “R” (version 3.1.3 (2015–03-09)) [45]. Generally, colony reproductive output of phase II was compared. Data were normally distributed for the number of eggs and brood items, number of hatched workers and sexuals, weight as well as days until the first offspring hatched (Shapiro-Wilk normality tests, *p* > 0.05). Timespan until the first sexuals eclosed and foraging activity were not normally distributed. Offspring number, development and weight were analyzed by the nonparametric PerMANOVA [46] for the effect of queen and worker age and to include the effect of the source colony and the mean number of workers present per colony per scan throughout the duration of phase II. Pairwise comparisons were done by two-tailed Welch Two Sample t-tests in the case of parametric data and Mann-Whitney U-tests in the case of non-parametric data.

Queen and worker survival was compared by Kaplan-Meier Survival Analysis using the package “survival” [47]. Differences between groups were compared by pairwise survival tests with control for a false discovery rate (Benjamini & Hochberg, function “BH”) [48]. As several workers escaped from the experimental nest boxes, only workers whose death could be verified were used for the analysis. The recorded number of deaths therefore differs from the actual number of workers in the experiment. In two colonies, queens escaped from the nest boxes, hence we included these dates as censored data.

## Results

### (a) offspring number

**Fig. 2:**
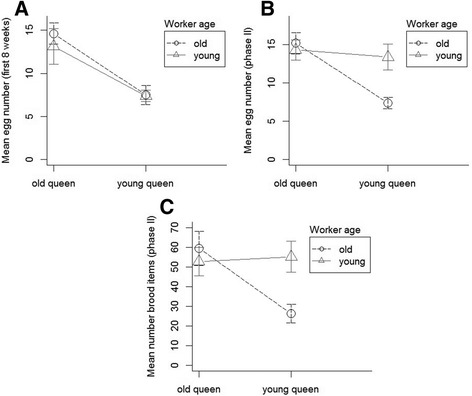
Brood production in colonies of the ant *Cardiocondyla obscurior* with different age compositions (mean, 95% CI). **a** Mean number of eggs produced during the first eight weeks after colony establishment and **b**) between colony establishment and the queen death, dependent on queen and worker age (12 weeks old vs. freshly eclosed when the colonies were set up). **c** Mean number of brood (eggs, larvae, pupae) produced between colony establishment and queen death, dependent on queen and worker age

When placed in a new nest without brood, young queens laid their first eggs almost two weeks later than 12 weeks old queens with previous reproductive experience (Mann-Whitney U-Test: W = 0.5, *p* = 0.0001; *n* = 19, mean days ± sd: old queens 2.27 ± 0.86; young queens 14.75 ± 5.61). Consequently, young queens produced fewer eggs than old queens during the first eight weeks after colony set-up (Welch Two Sample t-test: *t* = 4.8; df = 13.4, *p* = 0.0003, Fig. 2a). Yet, mean egg laying rates over the whole duration of phase II differed not significantly between old and young queens (Welch Two Sample t-test: *t* = 2.03, df = 11.87, *p* = 0.065). Over the whole period II (between colony set-up and queen death, see Material and Methods), queen egg laying rate was significantly influenced by queen age and by the interaction between queen and worker age (PerMANOVA, queen age: F = 6.4, df = 1, *p* = 0.027; worker age: F = 2.3, df = 1, *p* = 0.154; interaction: F = 6.8, df = 1, *p* = 0.02; average number of workers present per scan: F = 0.9, df = 1, *p* = 0.36; stock colony: F = 0.37, df = 4, *p* = 0.8 Fig. 2 b): old queens laid more eggs, and queens laid more eggs when cared for by workers of similar age.

The mean number of brood items (eggs, larvae, pupae) produced during phase II did not differ among the four groups (PerMANOVA: F = 2.6, df = 3, *p* = 0.09) but was significantly affected by the interaction between queen and worker age (PerMANOVA, queen age: F = 2.4, df = 1, *p* = 0.14; worker age: F = 1.6, df = 1, *p* = 0.26; interaction: F = 6.3, df = 1, *p* = 0.02; worker number: F = 1.6, df = 1, *p* = 0.23; stock colony: F = 0.83.4; df = 4, *p* = 0.5 Fig. 2c).

### (b) developmental time

**Fig. 3:**
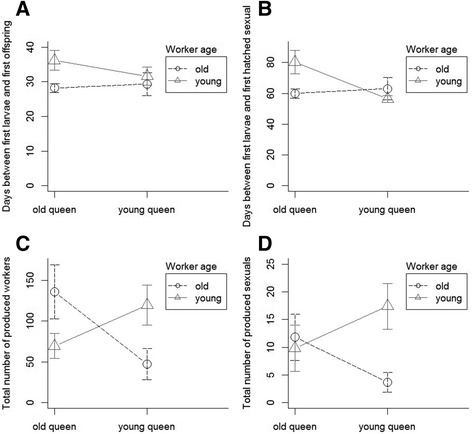
Timing and success of brood production of *Cardiocondyla obscurior* colonies with different combinations of old and young queens and workers (mean, 95% CI). **a** Days between first larvae and first hatched worker. **b** Days between first larvae and first hatched sexual offspring. **c** Total number of produced workers. **d** Total number of produced sexuals

The developmental time of the brood depended on worker age and was additionally affected by worker number (PerMANOVA, queen age: F = 0.5, df = 1, *p* = 0.5; worker age: F = 5.05, df = 1, *p* = 0.049; interaction: F = 0.04, df = 1, *p* = 0.9; worker number: F = 6.4, df = 1, *p* = 0.028, stock colony: F = 0.5, df = 4, *p* = 0.76, Fig. 3a). Old workers reared the queens’ first offspring faster than young workers did (Welch Two Sample t-test: *t* = −2.3, df = 16.36, *p* = 0.037). Colonies with old queens produced the first sexual offspring earlier than colonies with young queens (Mann-Whitney-U-Test: W = 63, *p* = 0.045). Furthermore, the production of the first sexual offspring depended on the queen age and the worker number inside the nest (PerMANOVA, queen age: F = 6.6, df = 1, *p* = 0.029; worker age: F = 2.2, df = 1, *p* = 0.17; interaction: F = 1.0, df = 1, *p* = 0.34; worker number: F = 9.6, df = 1, *p* = 0.015, stock colony: F = 1.1, df = 4, *p* = 0.4, Fig. 3b).

Colonies with old queens and young workers or vice versa (OQYW and YQOW) produced fewer worker offspring than colonies in which workers and queens were of similar age (OQOW and YQYW), as worker offspring was affect by the interaction between queen and worker age (PerMANOVA, queen age: F = 0.09, df = 1, *p* = 0.89; worker age: F = 0.26, df = 1, *p* = 0.73; interaction: F = 5.6, df = 1, *p* = 0.02, worker number: F = 0.3, df = 1, *p* = 0.69, stock colony: F = 1.3, df = 4, *p* = 0.3, Fig. 3 c). The number of produced sexual offspring was affected by the interaction between queen and worker age (PerMANOVA, queen age: F = 0.2, df = 1, *p* = 0.96; worker age: F = 0.5, df = 1, *p* = 0.68; interaction: F = 3.2, df = 1, *p* = 0.04; worker number: F = 1.01, df = 1, *p* = 0.4; stock colony: F = 1.4, df = 4, *p* = 0.2, Fig. 3 d).

### (c) offspring weight

The wet weight of the first worker offspring depended on queen age and on its interaction with worker age (PerMANOVA, queen age: F = 15.3, df = 1, *p* = 0.03; worker age: F = 1.6, df = 1, *p* = 0.3; interaction: F = 13.9, df = 1, *p* = 0.028; worker number: F = 0.13, df = 1, *p* = 0.78; stock colony: F = 0.96, df = 4, *p* = 0.5). The first workers produced by young queens were lighter than those produced by old queens (Welch Two Sample t-test: *t* = 2.88, df = 8.18, *p* = 0.02). In particular, the offspring produced by young queens and nursed by old workers was very light (Tukey HSD: YQOW vs. OQOW *p* = 0.01, other comparisons n. s.). The weight of the later-produced workers was affected by worker age and the worker number (PerMANOVA, queen age: F = 2.4, df = 1, *p* = 0.22; worker age: F = 12.6, df = 1, *p* = 0.036; interaction: F = 0.85, df = 1, *p* = 0.4; worker number: F = 12.9, df = 1, *p* = 0.03), with colonies containing more workers producing heavier offspring.

### (d) behavioral observations

Scan sampling suggested that the workers’ foraging activity during the first twelve days of colony founding depended on queen age. Independent of worker age, workers foraged always more in colonies with a young queen in comparison to colonies with old queens, with 30% and 17% foraging workers (Mann-Whitney U-Test: W = 61, *p*-value = 0.004), respectively (PerMANOVA, queen age: F = 9.5, df = 1, *p* = 0.0027; worker age: F = 2.07, df = 1, *p* = 0.15; interaction: F = 0.16, df = 1, *p* = 0.82, stock colony: F = 0.54, df = 5, *p* = 0.8).

**Fig. 4:**
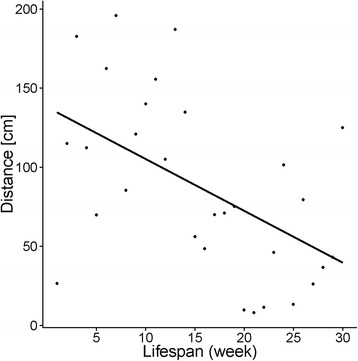
Distance [cm] covered by *C. obscurior* workers of different age during 10 min

Video analyses in additional colonies revealed that over the complete period of time, workers travelled less distance (Regression: R^2^ = 0.26, *p* < 0.001, Fig. 4) and spent more time immobile with age (Regression: R^2^ = 0.39, *p* = 0.0002). Regardless of queen and worker age, workers were observed foraging already during the first day (6 scans) of the experiment (OQOW 2nd scan, OQYW 2nd scan; YQOW 1st scan; YQYW 3rd scan).

### (e) queen and worker mortality

In our experiment, old queens reached slightly longer total life spans than young queens (Log-Rank-Test: χ^2^ = 3.5, df = 1, *p* = 0.06). Moreover, queens of the different treatment groups differed in their survival (Log-Rank-Test: χ^2^ = 8.9, df = 3, *p* = 0.03, Additional file 1: Figure S2). Old queens lived significantly longer with old (median queen lifespan: 35 weeks, Q1: 33, Q3: 35, Χ^2^ = 9.6, df = 1, *p* = 0.002) than with young workers (Χ^2^ = 9.6, df = 1, *p* = 0.002; OQYW median queen lifespan: 31 weeks, Q1: 30, Q3: 31) and also longer than young queens living with young workers (Χ^2^ = 7.5, df = 1, *p* = 0.006; YQYW median queen lifespan: 24 weeks, Q1: 23, Q3:27) (for details see Additional file 1: Table S1). It must be noted here that because of the failure of several set-ups (see Methods) sample size was low (OQOW *n* = 5; OQYW *n* = 6, YQOW *n* = 3; YQYW *n* = 5).

**Fig. 5:**
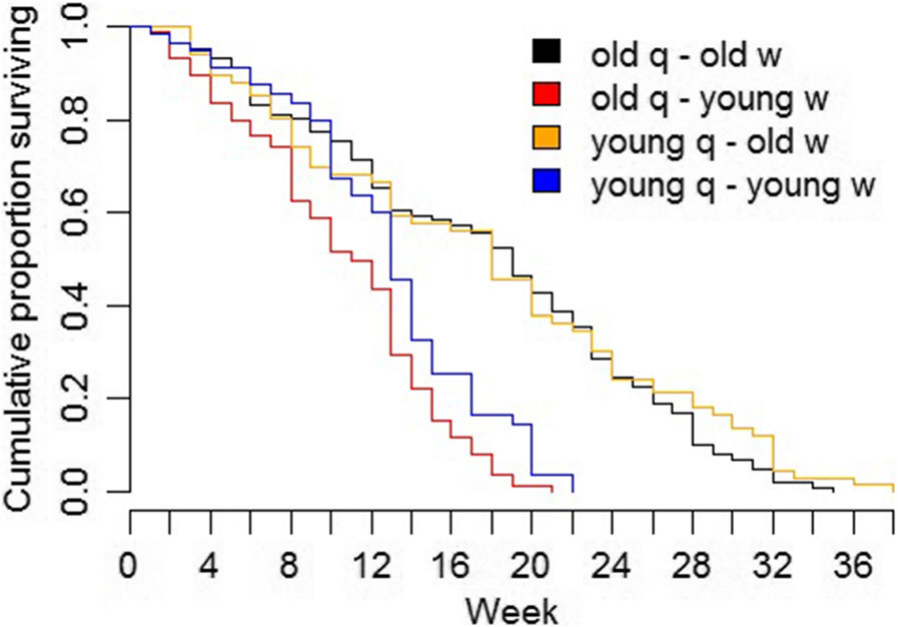
Survival of workers dependent on queen (q) and worker (w) age during the entire duration of the experiment (phase I + II)

Worker mortality over the entire experimental time (before colony set-up, i.e., phase I, and after colony set-up until queen death, phase II) was determined by the age of workers when the new colony was established. Workers, which had already been 12 weeks old at the beginning of phase II, lived significantly longer than workers, which had eclosed shortly before colony set-up (Log-Rank-Test: old vs. young workers, χ^2^ = 80.2, df = 1, *p* < 0.0001). During the first four weeks after the beginning of phase II only very few old workers died. The mortality rate of old workers at a chronological age of 12 to 16 weeks at the beginning of phase II was significantly lower than the mortality rate of young workers at a similar chronological age during phase II (Mann-Whitney U-Test: W = 0, *p* = 0.027). The proportion of dead workers in colonies with young workers in the first 12 weeks of phase II (0.30; period: 12 weeks) was the same as in the 12 weeks of phase I (0.32; period: 12 weeks) (Log-Rank-Test: χ^2^ = 0.14; df = 1, *p* > 0.5). This suggests that the physiological age of old workers was more or less set to zero when transferred into a novel situation of colony founding, i.e., with a new queen and without brood. Additionally, worker mortality was affected by the composition of the colony: young workers live longer when they are together with young queens, while old workers live longer with old queens (Fig. 5) (Log-Rank-Test: χ^2^ = 75.3, df = 3, *p* < 0.0001; for details see Additional file 1: Table S2).

Worker survival and the number of larvae in a colony were correlated (old workers: rho = 0.7, *p* < 0.0001, young workers: rho = 0.7, *p* < 0.0001), probably reflecting the fact that worker mortality as well as larval number in the colonies increased with time. Similarly, worker mortality and the number of newly eclosed workers were correlated (old workers: rho = 0.5, *p* < 0.01, young workers: rho = 0.7, *p* < 0.0001).

## Discussion

We investigated how the chronological age of queens and workers affects reproductive success and the individual speed of aging in the ant *Cardiocondyla obscurior*. Matching previous observations [49], old queens did not show any signs of reproductive senescence. They initially laid more eggs after colony set-up than young queens, but the latter caught up with time. Both old and young workers were able to perform all necessary tasks, and colonies produced similar numbers of offspring independent of worker age. This supports the view that task allocation in manipulated colonies of *C. obscurior* is not strictly age-based but relatively plastic [33], which is consistent with the repertoire expansion model [18].

Nevertheless, colony performance depended on queen and worker age and especially on the interaction between these two factors. Furthermore, the colony age structure had a strong consequence for the residual life spans of individuals: queens lived two to four weeks longer with old than with young workers, and workers, which were 12 weeks old when starting a new colony, outlived workers, which had eclosed shortly before colony founding, by five to eight weeks. Aging in ants therefore appears to depend less on chronological age but on their tasks and the status of the colony, as has previously been demonstrated in honey bee workers [26, 35–37].

Queen age determined the initial growth rate of the newly founded colony, while the age composition of queens and workers gained importance as the colony matured. Colonies with queens and workers of similar age appeared to perform better than colonies in which they were of different age.

### Reproductive success

The egg laying rate of *C. obscurior* queens increases with age [49, 50]. This explains why in our study old queens laid more eggs than young queens at the beginning of the experiment. Additionally, old queens appeared to produce eggs of a higher “quality,” as suggested by higher individual weight of workers produced by old queens. Young ant queens need some time to switch on their reproductive apparatus after mating, and at least queens, which independently found new colonies, invest fewer resources in their first offspring than old, established queens (“nanitic” workers, e.g. [51]). Worker age gains importance when the brood develops as workers are responsible for supplying larvae with food. The brood care experience older workers obtained during phase I might have contributed to the faster development of brood and the increased weight of the first produced worker offspring in colonies with old workers. This supports the hypothesis that workers expand their task repertoire [18, 34]. Nevertheless, colonies with queens and workers being of the same age (OQOW, YQYW) grew faster and managed to rear more worker offspring. In any case, our data show that old workers are still well capable of efficiently caring for the brood, as previously shown in other social insects [26, 52, 53]; but see [54].

Colonies with old queens and a high worker number produced first sexual offspring most rapidly. However, the total reproductive output was determined by the interaction between queen and worker age, which was reflected by the high number of sexual offspring in young colonies with young queens and young workers.

### Worker mobility

Freshly eclosed workers of *C. obscurior* were capable of adjusting their behavior to the colony needs and to become foragers. In contrast, young workers of *Pheidole dentata* have been suggested to be behaviorally immature and to be unable to take over new tasks [55]. Young workers of *C. obscurior* left the nest shortly after eclosion and started to forage already during the first few days of our experiment. Their higher mobility and the slower development of larvae in their colonies might reflect their inexperience or developmental immaturity [55, 56]. Independent of their own age, workers foraged more frequently in colonies with young queens, which probably needed more resources to develop their ovarioles and to increase their egg laying rate than old queens.

### Queen and worker lifespan

The age composition of colonies affected the lifespan of both queens and workers. Old queens reached the highest lifespan when living with old workers. This might be a consequence of the rapid decline of colonies with young workers, which had a much shorter residual life expectancy than old workers. In our experimental colonies without old workers, young workers reached median total lifespans of 11 to 13 weeks, i.e., 2 to 3 weeks less than reported for workers in unmanipulated colonies [43]. In contrast, in colonies consisting of only old workers they outlived “normal” workers by 3 to 4 weeks and the young workers in our experimental colonies by more than 6 weeks.

The OQYW and YQYW treatments of our experiment forced young workers to forage prematurely, which may have accelerated their senescence. Previous studies in honey bees have revealed a negative effect on longevity caused by an earlier onset of foraging [26, 27, 57, 58]. In contrast, in the OQOW and YQOW treatments at least a few old workers had to reverse their tasks and return to brood care. Here, the average mortality rate of old workers stagnated during the first weeks after the colonies were set-up. This clearly documents that the aging trajectory of social insect workers does not depend on their chronological age. Instead, their behavioral status is more important and the transition from foraging to nursing reverses the pattern of aging and senescence (e.g. [17, 52, 59–61]).

While previous studies rarely monitored the exact age of foragers when they reverted to nursing [62], in our experiment all workers in OQOW and YQOW colonies were 12 weeks old at the beginning of phase II, i.e., reversal did not only occur among very young foragers. We can exclude an influence of colony size on worker lifespan as reported for the ant *Lasius niger* and honeybees [58, 63], as worker pupae for the set ups were taken from fully established colonies and worker number was standardized at the beginning of the experiment. Furthermore, at least in honeybees and the ant *Platythyrea punctata*, the reversal to nursing is associated with increased ovarian development [52, 61] and hence physiological changes. Thus, proximately, task and social environment may lead to changes in hormone and vitellogenin titers, which again may affect senescence (e.g. [64, 65]). In contrast, *Cardiocondyla* workers completely lack ovaries and the reversion to nursing does not alter their “reproductive” status. Still, changes in hormone status are likely to occur but have to be verified in the future [64, 65].

Ultimately, the observed plasticity of aging and division of labor in *C. obscurior* might increase the success of colony founding by budding, i.e., the joint emigration of queens and workers from the natal nest. This process likely changes the age structure of the old and the new colony and requires considerable flexibility in task allocation independent of age.

## Conclusions

In summary, our data show that colonies of *C. obscurior*, in which all workers are of similar age, develop well, regardless of worker age, but that the interaction between queen age and worker age may have a strong effect on colony productivity. Old queens had a higher reproductive output when starting a new colony together with old workers. While worker age had slight effects on queen longevity, exposing old workers to the conditions of colony founding set their internal clocks back and increased their life spans by 50%. This highlights the enormous plasticity of aging and senescence in social insects and the resilience of division of labor to extreme changes in social organization.

## Additional file

Additional file 1:
**Figures. S1 & S2**, **Tables S1 & S2**. (DOCX 30 kb)
Additional file 2: Raw Data S3. (XLSX 22 kb)
