# Supplementary material for: Group demography affects ant colony performance and individual speed of queen and worker aging
Source: BMC Evol Biol. 2017 Aug 1;17:173. doi: 10.1186/s12862-017-1026-8 (PMC5540184; doi:10.1186/s12862-017-1026-8)
Supplement: Additional file 1: — Figures. S1 & S2, Tables S1 & S2. (DOCX 30 kb) [file 12862_2017_1026_MOESM1_ESM.docx]

**Additional file 1**

**
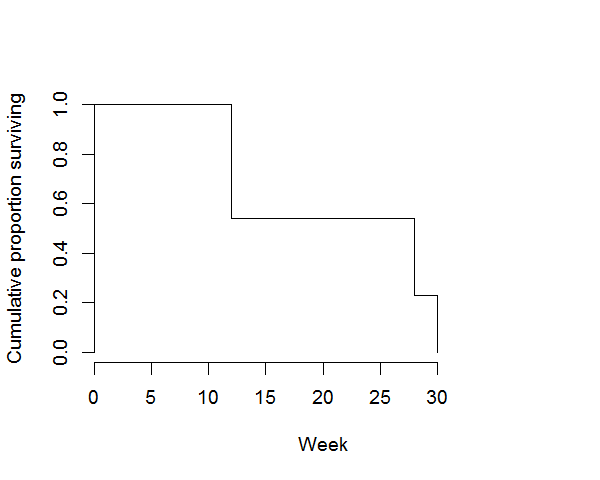
**

Figure S1, Worker survival rate during videotaped behavioral observations


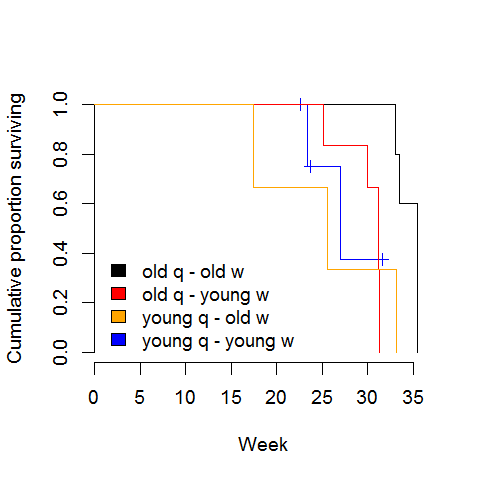


Figure S2, Survival of queens dependent on queen (q) and worker (w) age during the entire duration of the experiment (phase I + II).

Table S1, Pairwise comparison data of queen mortality over the entire experimental time. Significant values marked bold.

|  | Median lifespan in weeks (quartiles) | YQYW | | OQOW | | OQYW | |
| --- | --- | --- | --- | --- | --- | --- | --- |
| OQOW, n = 5 | 35  (33, 35) | **p = 0.03** |  | |  | |  |
| OQYW, n = 6 | 31  (30, 31) | p = 0.25 | **p = 0.03** | |  | |  |
| YQOW, n = 3 | 26  (22, 29) | p = 1.0 | | p = 0.13 | | p = 0.84 | |
| YQYW, n =3 | 24  (23, 27) |  | |  | |  | |

Table S2, Worker mortality according to colony composition over the entire experiment. Significant values marked bold.

|  | Median lifespan in weeks (quartiles) | YQYW | OQOW | OQYW |
| --- | --- | --- | --- | --- |
| OQOW, n =101 | 19  (11, 24) | **p = 0.002** |  |  |
| OQYW, n = 85 | 11  (7, 14) | **p = 0.016** | **p < 0.0001** |  |
| YQOW, n = 66 | 18  (8, 24) | **p = 0.015** | p = 0.95 | **p < 0.0001** |
| YQYW, n = 55 | 13  (10, 17) |  |  |  |
